# Efficient Production of (*R*)-2-Hydroxy-4-Phenylbutyric Acid by Using a Coupled Reconstructed d-Lactate Dehydrogenase and Formate Dehydrogenase System

**DOI:** 10.1371/journal.pone.0104204

**Published:** 2014-08-04

**Authors:** Binbin Sheng, Zhaojuan Zheng, Min Lv, Haiwei Zhang, Tong Qin, Chao Gao, Cuiqing Ma, Ping Xu

**Affiliations:** 1 State Key Laboratory of Microbial Technology, Shandong University, Jinan, People's Republic of China; 2 College of Chemical Engineering, Nanjing Forestry University, Nanjing, People's Republic of China; Louisiana State University and A & M College, United States of America

## Abstract

**Background:**

(*R*)-2-Hydroxy-4-phenylbutyric acid [(*R*)-HPBA] is a key precursor for the production of angiotensin-converting enzyme inhibitors. However, the product yield and concentration of reported (*R*)-HPBA synthetic processes remain unsatisfactory.

**Methodology/Principal Findings:**

The Y52L/F299Y mutant of NAD-dependent d-lactate dehydrogenase (d-nLDH) in *Lactobacillus bulgaricus* ATCC 11842 was found to have high bio-reduction activity toward 2-oxo-4-phenylbutyric acid (OPBA). The mutant d-nLDH^Y52L/F299Y^ was then coexpressed with formate dehydrogenase in *Escherichia coli* BL21 (DE3) to construct a novel biocatalyst *E. coli* DF. Thus, a novel bio-reduction process utilizing whole cells of *E. coli* DF as the biocatalyst and formate as the co-substrate for cofactor regeneration was developed for the production of (*R*)-HPBA from OPBA. The biocatalysis conditions were then optimized.

**Conclusions/Significance:**

Under the optimum conditions, 73.4 mM OPBA was reduced to 71.8 mM (*R*)-HPBA in 90 min. Given its high product enantiomeric excess (>99%) and productivity (47.9 mM h^−1^), the constructed coupling biocatalysis system is a promising alternative for (*R*)-HPBA production.

## Introduction

(*R*)-2-hydroxy-4-phenylbutyric acid [(*R*)-HPBA] and ethyl (*R*)-2-hydroxy-4-phenylbutyrate [(*R*)-HPBE] can be used as the key precursors for the production of angiotensin-converting enzyme (ACE) inhibitors [1]–[5]. ACE inhibitors such as benazepril, enalapril, lisinopril, ramipril, and quinapril are widely used in the first-line therapy of hypertension and congestive heart failure [6]–[9]. Owing to the substantial demand for these drugs, various chemical or biological processes have been developed to produce (*R*)-HPBA or (*R*)-HPBE. In recent years, great success has been achieved in asymmetric synthesis of (*R*)-HPBE catalyzed by recombinant reductases [10], [11]. For example, whole cells of a recombinant *Escherichia coli* strain harboring CgKR2 and glucose dehydrogenase (GDH) were applied in preparing (*R*)-HPBE with high concentration, desirable enantiomeric excess (ee) (>99%) and yield [10]. Compared with that of (*R*)-HPBE, the product yield and concentration of the reported (*R*)-HPBA synthesis processes remained unsatisfactory [1], [12].

In previous studies, enzymatic resolution and asymmetric reduction were used in the biological production of (*R*)-HPBA. Compared with enzymatic resolution catalyzed by hydrolases, especially lipases [4], [9], [13], asymmetric bio-reduction of 2-oxo-4-phenylbutyric acid (OPBA) by dehydrogenases is more desirable because of its excellent stereoselectivity and high theoretical yield up to 100% [1], [14]. For practical production of (*R*)-HPBA from OPBA through bio-reduction, highly efficient reductases and cofactor regeneration systems are needed.

In contrast to the (*R*)-HPBE preparation processes, which often utilize a specific carbonyl reductase, the production of (*R*)-HPBA from OPBA is catalyzed by 2-ketoacid reductases, especially NAD-dependent d-lactate dehydrogenase (d-nLDH) [12], [15]. However, as an unnatural substrate of d-nLDH, OPBA could not be efficiently catalyzed by the biocatalyst because of its large aromatic group at C-4.On the other hand, cofactor regeneration systems that utilize glucose as a co-substrate in (*R*)-HPBE production may not be the proper choice in the (*R*)-HPBA production. The addition of glucose to the reaction system may result in the production of organic acids (such as gluconic acid and lactic acid) as byproducts and increase the complexity of the (*R*)-HPBA separation process [16], [17]. In a previous study, a partially purified d-nLDH was used to transform OPBA to (*R*)-HPBA. The cofactor NADH was regenerated by formate dehydrogenase (FDH) present in whole cells of *Candida boidinii* ATCC 32195. Although this NADH regeneration system produced CO_2_ as the only byproduct, which facilitated the isolation of (*R*)-HPBA, the whole cells of *C. boidinii* should be pre-treated with toluene to make them permeable [12].

**Figure 1:**
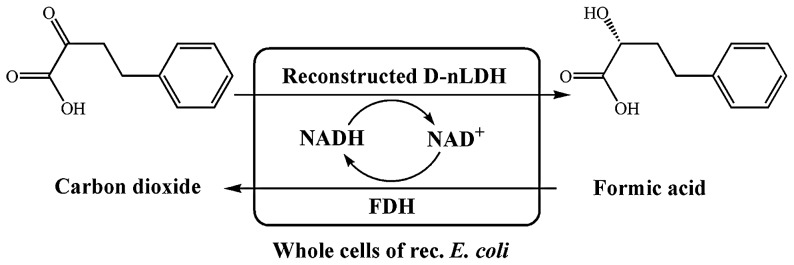
Scheme for (*R*)-HPBA production from OPBA by using a coupled system of reconstructed d-nLDH and FDH.

In our previous studies, the d-nLDH in *Lactobacillus bulgaricus* ATCC 11842 was rationally re-designed and then used for the bio-reduction of substrates with large aliphatic or aromatic groups at C-3 [14]. In this study, the activities of different d-nLDH mutants toward OPBA (2-oxo carboxylic acids with an aromatic group at C-4) were assayed. The most active reconstructed d-nLDH was co-expressed with FDH from *C. boidinii* NCYC 1513 in *E. coli* BL21 (DE3). Then, a novel process utilizing whole cells of recombinant *E. coli* was developed for efficient production of (*R*)-HPBA from OPBA (Fig. 1).

## Materials And Methods

### Materials

OPBA was purchased from Gracia Chemical Technology Co., Ltd. Chengdu (China). Isopropyl-β-d-1-thiogalactopyranoside (IPTG), phenylmethanesulfonyl fluoride (PMSF), and (*R*)-HPBA were purchased from Sigma-Aldrich. (*S*)-HPBA was purchased from J&K Chemical. All other chemicals in this study were of reagent grade.

### Microorganisms And Growth Conditions

The bacterial strains, plasmids, and oligonucleotide primers used in this study are listed in Table 1. *E. coli* DH5α and BL21 (DE3) were used for general cloning and expression procedures, respectively. *E. coli* WD, *E. coli* D1, and *E. coli* D2 were used to express wild d-nLDH, d-nLDH^F299Y^, and d-nLDH^Y52L/F299Y^, respectively [14]. *E. coli* PD containing the vector pETDuet-1 was used as a control. The plasmid pETDuet-*ldhD* ^Y52L/F299Y^-*fdh* was constructed as follows: the *ldhD* ^Y52L/F299Y^ gene was amplified using primers D.f and D.r with plasmid pETDuet-*ldhD* ^Y52L/F299Y^ as a template. The *fdh* gene was amplified using primers F.f and F.r with genomic DNA of *C. boidinii* NCYC 1513 as a template. The resulting PCR products *ldhD* ^Y52L/F299Y^ and *fdh* were digested with *Nco*I-*Bam*HI and *Nde*I-*Xho*I, respectively, and cloned into MCS1 and MCS2 of pETDuet-1 successively to construct pETDuet-*ldhD* ^Y52L/F299Y^-*fdh*. The plasmid pETDuet-*ldhD* ^Y52L/F299Y^-*fdh* was then transformed into *E. coli* BL21 (DE3) to construct *E. coli* DF. All of the *E. coli* strains were grown in Luria-Bertani (LB) medium, and ampicillin was added at a concentration of 100 µg m1^−1^ if necessary.

**Table 1:** Strains, plasmids, and oligonucleotide primers used in this study.

Strain, plasmid, or primer	Relevant characteristics	Source or reference
Strain		
*E. coli* DH5α	φ80 *lacZ*ΔM15 Δ(*lacZ*YA-*arg*F) U169 *recA*1 *endA*1 *hsdR*17 *supE*44λ-*thi*-1	Invitrogen
*E. coli* BL21(DE3)	*F^−^ ompT gal dcm lon hsdS*_B_(r_B_^−^m_B_^−^) *λ*(*DE3*)	Novagen
*C. boidinii* NCYC 1513	Wild-type, source of *fdh* gene	NCYC^a^
*E. coli* PD	*E. coli* BL21(DE3) containing vector pETDuet-1	This study
*E. coli* WD	*E. coli* BL21(DE3) expressing wild type d-nLDH	[14]
*E. coli* D1	*E. coli* BL21(DE3) expressing d-nLDH^F299Y^	[14]
*E. coli* D2	*E. coli* BL21(DE3) expressing d-nLDH^Y52L/F299Y^	[14]
*E. coli* DF	*E. coli* BL21(DE3) expressing d-nLDH^Y52L/F299Y^ and FDH	This study
Plasmid		
pETDuet-1	Expression vector, Amp^r^	Novagen
pETDuet-*ldhD* ^Y52L/F299Y^	N-terminal His-tagged *ldhD* ^Y52L/F299Y^ gene in pETDuet-1	[14]
pETDuet-*ldhD* ^Y52L/F299Y^-*fdh*	Both *ldhD* ^Y52L/F299Y^ and *fdh* without His-tag in pETDuet-1	This study
Oligonucleotide primer	Sequence (5′→3′)	
D.f	CCATGGTGACTAAAATTTTTGCTTACGCA (*Nco*I)	
D.r	GGATCCTTAGCCAACCTTAACTGGAGTTT (*Bam*HI)	
F.f	CATATGAAGATCGTTTTAGTCTTATATGATGCTGGTA (*Nde*I)	
F.r	CTCGAGTTATTTCTTATCGTGTTTACCGTAAGCTTTG (*Xho*I)	

a NCYC, National Collection of Yeast Cultures.

doi:10.1371/journal.pone.0104204.t001

### Biocatalyst Preparation

The recombinant strains of *E. coli* PD, *E. coli* WD, *E. coli* D1, *E. coli* D2, and *E. coli* DF were all cultured in LB medium (100 µg ml^−1^ ampicillin) at 37°C to an optical density of 0.6 at 600 nm. IPTG (1 mM) was then added to induce protein expression, and cultures were grown at 16°C for a further 12 h. Cells were harvested by centrifugation at 6,000 rpm for 10 min, washed twice with 67 mM phosphate buffer solution (pH 7.4), and then subjected to successive biotransformation.

### Optimization Of Biocatalysis Conditions

To optimize the biotransformation conditions, 5-ml reaction mixtures were incubated at 37°C and 120 rpm in a 25-ml flask. The pH was adjusted from 5.5 to 8.5. The concentrations of OPBA and formate were 25–175 mM. The concentration of the whole cells was 1–8 g dry cell weight (DCW) l^−1^. Samples (0.2 ml) were collected periodically and centrifuged at 12,000 rpm. The concentrations of OPBA and (*R*)-HPBA in the supernatant were analyzed by a high-performance liquid chromatography (HPLC) system (Agilent 1100 series, Hewlett-Packard, USA).

### Analytical Procedures

Cells of *E. coli* PD, *E. coli* WD, *E. coli* D1, *E. coli* D2, and *E. coli* DF were harvested, suspended in 67 mM phosphate buffer solution (pH 7.4) containing 1 mM PMSF, and then disrupted by sonication (Sonics 500 W; 20 KHz) for 5 min in an ice bath. Thereafter, intact cells and cell debris were removed by centrifugation, and the resultant crude extracts were subjected to successive d-nLDH activity assays. The reduction activities of d-nLDH wild-type and mutants toward OPBA were assayed at 37°C in 1 ml of 50 mM Tris-HCl buffer (pH 7.5) containing 0.2 mM NADH, 10 mM OPBA, and the crude extracts of *E. coli* PD, *E. coli* WD, *E. coli* D1, *E. coli* D2, and *E. coli* DF. The rate of NADH decrease was determined by measuring the absorbance change at 340 nm [14],[18],[19]. One unit of d-nLDH activity was defined as the amount that catalyzed the oxidation of 1 µmol of NADH per minute. The protein concentration was determined by the Lowry procedure by using bovine serum albumin as the standard [20].

OPBA and (*R*)-HPBA were measured by HPLC (Agilent 1100 series) equipped with an Agilent Zorbax SB-C18 column (150×4.6 mm, 5 µm) and a variable-wavelength detector at 210 nm. The mobile phase consisted of 1 mM H_2_SO_4_ and acetonitrile with a ratio of 85∶15 (v/v) at a flow rate of 0.7 ml min^−1^ at 30°C. Stereoselective assays for (*R*)-HPBA and (*S*)-HPBA were performed by HPLC analysis by using a chiral column (MCI GEL CRS10W, Japan) and a tunable UV detector at 254 nm. The mobile phase was 2 mM CuSO_4_ and acetonitrile with a ratio of 85∶15 (v/v) at a flow rate of 0.5 ml min^−1^ and a temperature of 25°C. The ee of (*R*)-HPBA was defined as [((*R*)-HPBA−(*S*)-HPBA)/((*R*)-HPBA+(*S*)-HPBA)]×100%.

## Results And Discussion

### Activity Of d-Nldh Wild-Type And Mutants Toward Opba

**Figure 2:**
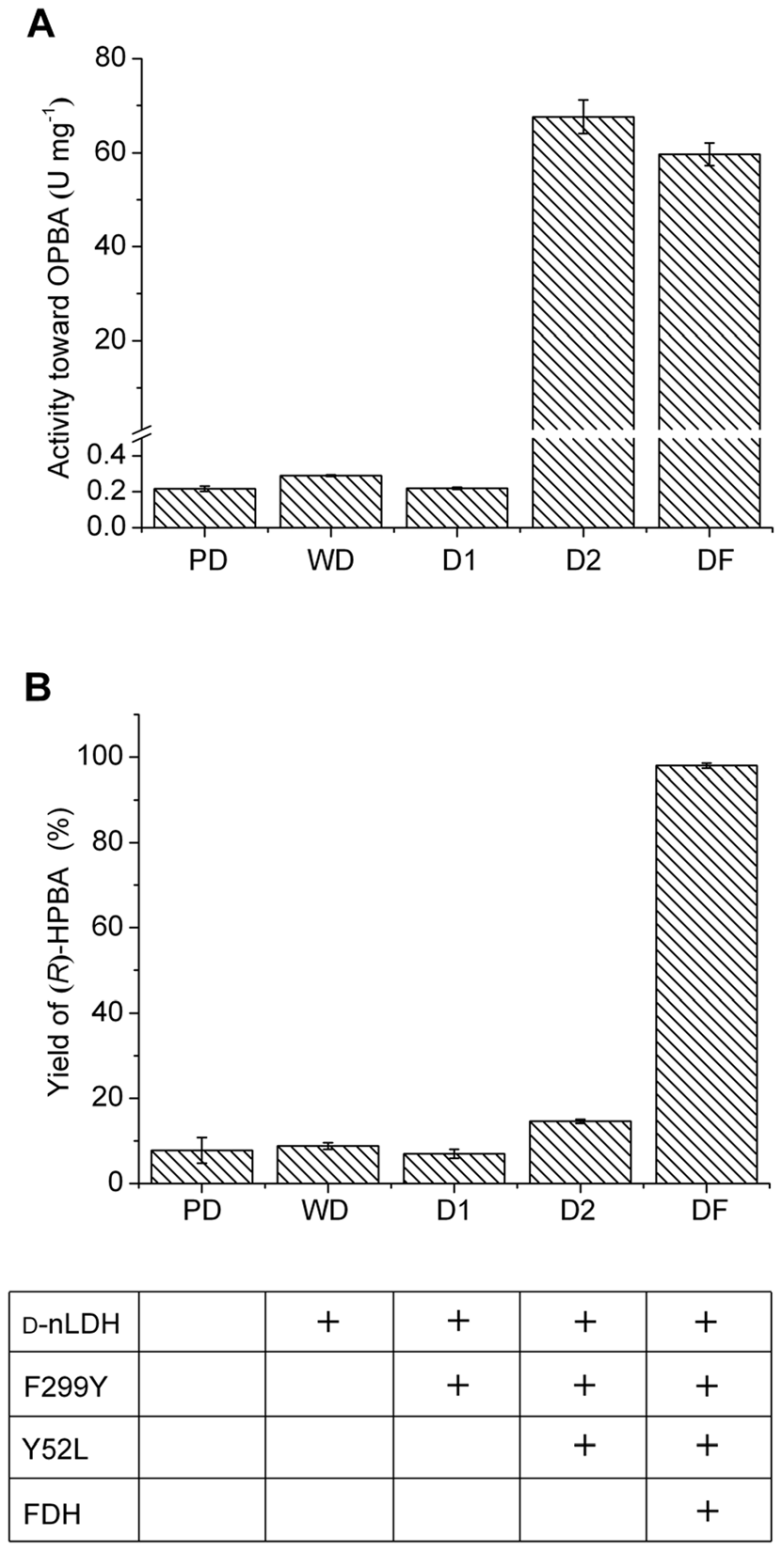
Feasibility of (*R*)-HPBA production through cofactor regeneration by reconstructed d-nLDH and FDH. (A) OPBA reduction activities in the crude extract of different *E. coli* strains. (B) Asymmetric reduction of OPBA by whole cells of different *E. coli* strains. For *E. coli* PD, *E. coli* WD, *E. coli* D1, and *E. coli* D2, NADH regeneration was conducted by the direct addition of 50 mM glucose. For *E. coli* DF, formate of 50 mM was added in the reaction broth for NADH regeneration.

To evaluate the possibility of transforming OPBA into (*R*)-HPBA by d-nLDH, the wild type d-nLDH from *L. bulgaricus* ATCC 11842 and its mutants were overexpressed in *E. coli* BL21 (DE3). Crude extracts of *E. coli* PD, *E. coli* WD, and *E. coli* D1 exhibited rather low OPBA reduction activity (Fig. 2A). The Y52L/F299Y mutant of d-nLDH caused the specific activity of the crude extract of *E. coli* D2 to be 233.2–312.3 fold higher than that in extracts of *E. coli* PD, *E. coli* WD, and *E. coli* D1. These results suggest that the mutant d-nLDH^Y52L/F299Y^ is rather active toward OPBA and may have the potential to efficiently produce (*R*)-HPBA from OPBA.

### Feasibility Of (*R*)-Hpba Production Through The Cofactor Regeneration System

Asymmetric reduction of OPBA by whole cells of *E. coli* PD, *E. coli* WD, *E. coli* D1, *E. coli* D2, and *E. coli* DF was investigated to further explore the potential by using d-nLDH in the synthesis of (*R*)-HPBA. OPBA at 50 mM was used as the substrate. Whole cells of *E. coli* PD, *E. coli* WD, *E. coli* D1, and *E. coli* D2 at a concentration of 8 g DCW l^−1^ were added to the reaction broth. The reaction was conducted at 37°C for 2 h. Here, NADH was regenerated through the direct addition of 50 mM glucose in the reaction system. Whole cells of *E. coli* D2 exhibited higher (*R*)-HPBA producing capability than did cells of *E. coli* PD, *E. coli* WD, and *E. coli* D1(Fig. 2B). However, the (*R*)-HPBA productivity (3.7 mM h^−1^) was still rather low because of the low efficiency of the NADH regeneration system. Additionally, organic acids, including pyruvic acid, lactic acid, and acetic acid, accumulated in the reaction broth (Fig. S1).

FDH is a good choice for NADH regeneration in a biocatalysis system because its substrate, formate, has a low cost and its product, carbon dioxide, is easily separated [21]–[25]. In this work, FDH was coexpressed with d-nLDH^Y52L/F299Y^ in *E. coli* DF and the (*R*)-HPBA production capability of the novel biocatalyst was investigated. Formate (50 mM) was added to the reaction broth for the regeneration of NADH. Although the activity of d-nLDH^Y52L/F299Y^ in the crude extract of *E. coli* DF was lower than in the extract of *E. coli* D2, whole cells of *E. coli* DF exhibited much higher (*R*)-HPBA producing capability than other biocatalysts (Fig. 2A and Fig. 2B). (*R*)-HPBA at 49.0 mM was obtained from 50 mM OPBA. The productivity of (*R*)-HPBA was 24.5 mM h^−1^. Thus, whole cells of *E. coli* DF were selected as biocatalysts for (*R*)-HPBA production in the subsequent experiments.

### Optimization Of Biocatalysis Conditions

**Figure 3:**
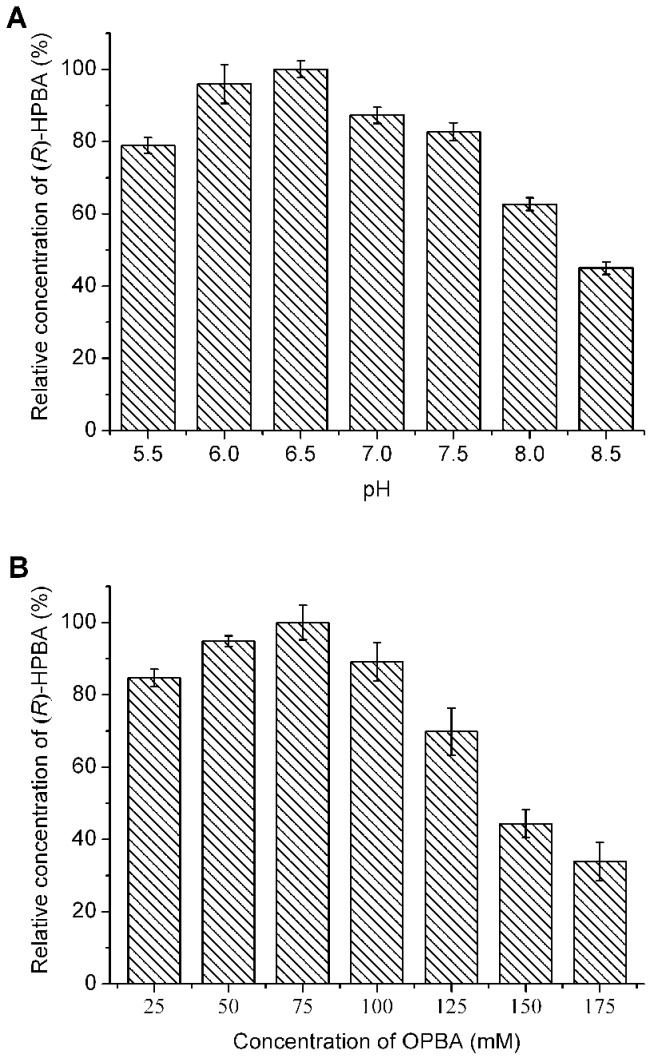
Optimization of the biocatalysis conditions. (A) pH. (B) Concentration of OPBA.

To achieve a higher product concentration, the biocatalytic conditions for (*R*)-HPBA production from OPBA by using whole cells of *E. coli* DF were optimized. The influence of the reaction pH was determined in reaction mixtures containing 13 g DCW l^−1^ whole cells of *E. coli* DF, 50 mM OPBA, 50 mM sodium formate, and 200 mM phosphate buffer (pH ranging from 5.5 to 8.5). After bioconversion at 37°C for 15 min, the highest (*R*)-HPBA production was detected at pH 6.5 (Fig. 3A).

To determine the effect of the OPBA concentration, reactions with eight different OPBA and sodium formate concentrations (25, 50, 75, 100, 125, 150, and 175 mM) were conducted at pH 6.5 and 37°C for 30 min. The highest (*R*)-HPBA production was detected when 75 mM OPBA was used (Fig. 3B). The effect of the biocatalyst concentration was also investigated to determine the optimal range. The biotransformation was conducted with 75 mM OPBA, 75 mM sodium formate, 200 mM phosphate buffer (pH 6.5), and whole cells of *E. coli* DF at six different concentrations (1, 3, 5, 6, 7, and 8 g DCW l^−1^). When the reactions were conducted to approximate 80% theoretical yield, the highest specific productivity was observed at a biocatalyst concentration of 6 g DCW l^−1^ (Table 2).

**Table 2:** Effects of concentration of whole cells on biotransformation^a^.

Cell concentration (g DCW l^−1^)	1	3	5	6	7	8
Reaction time (min)	285	140	75	55	50	45
(*R*)-HPBA concentration (mM)	33.0	61.8	59.9	59.4	60.3	61.1
Productivity^b^ (mM min^−1^ g^−1^ DCW)	0.116	0.147	0.160	0.180	0.172	0.170

a Value is the average value of three separate assays.

b Productivity was calculated when the reaction was conducted to approximately 80% of the theoretical yield except for the reaction at 1 g DCW l^−1^ whole cells.

doi:10.1371/journal.pone.0104204.t002

### Production Of (*R*)-Hpba Under Optimal Conditions

**Figure 4:**
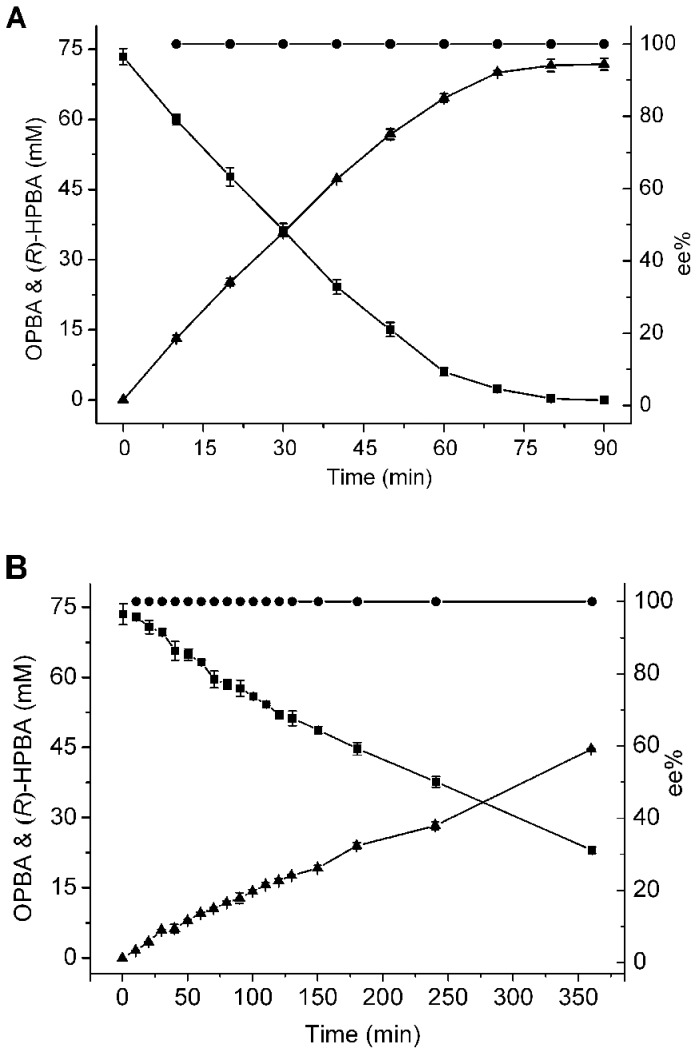
Time course of highly optically pure (*R*)-HPBA production from OPBA under optimal conditions. (A) Biotransformation using whole cells of *E. coli* DF as a biocatalyst and formate for cofactor regeneration. (B) Biotransformation using whole cells of *E. coli* D2 as a biocatalyst and glucose for cofactor regeneration. (▪), OPBA; (▴), (*R*)-HPBA; (•), ee.

On the basis of the results presented above, an optimal bioconversion system for production of optically pure (*R*)-HPBA from OPBA was developed. Biotransformation was conducted at 37°C in 200 mM phosphate buffer (pH 6.5) with 6 g DCW l^−1^ whole cells of *E. coli* DF as the biocatalyst. As shown in Fig. 4A, 71.8 mM (*R*)-HPBA with a high enantiomeric purity (ee >99%, Fig. S2) was obtained from 73.4 mM OPBA in 90 min. When whole cells of *E. coli* D2 only expressing d-nLDH^Y52L/F299Y^ were used as the biocatalyst, and glucose was added for NADH regeneration, only 44.7 mM (*R*)-HPBA was produced with a yield of 60.9% after 360 min (Fig. 4B).

Many biocatalysts have been used in the enantioselective production of (*R*)-HPBE and (*R*)-HPBA through bio-reduction [1],[12],[26]–[28]. Compared with (*R*)-HPBE production processes, the product concentrations of the reported (*R*)-HPBA synthesis processes were rather low (Table 3) [1],[10]–[12],[15],[29],[30]. In the previous study, purified d-LDH from *Staphylococcus epidermidis* and FDH from *Candida boidinii* were applied for (*R*)-HPBA production. (*R*)-HPBA at a concentration of 182 mM was produced, which is the highest reported yield of (*R*)-HPBA to date [15]. However, problems concerning the application of the process, such as the complicated enzyme purification and costly cofactor addition, remain. In the present work, mutant d-nLDH and FDH were co-expressed in *E. coli* DF and used for (*R*)-HPBA production from OPBA. The productivity (47.9 mM h^−1^) and ee (>99%) of the product were rather high for (*R*)-HPBA production. Additionally, given the simple composition of the biocatalytic system, separation of (*R*)-HPBA from the biocatalytic system would be relatively inexpensive. Therefore, the novel process established in this study could also be used as a promising route for the production of highly optically pure (*R*)-HPBA.

**Table 3:** Comparison of recently reported processes for (*R*)-HPBA or (*R*)-HPBE production through bio-reduction.

Biocatalyst	Product (mM)	Productivity (mM h^−1^)	ee (%)	Co-substrate	References
Whole cells of *Candida boidinii* CIOC21	20	1.7	99	5% glucose	[27]
Whole cells of *Bacillus pumilus* Phe-C3	29.6	1.1	97.1	2% glucose	[28]
Whole cells of *Candida krusei* SW2026	79.5	5.0	97.4	5% glucose	[26]
*Saccharomyces cerevisiae* pretreated with α-phenacyl chloride	4.8	0.2	92	-	[29]
*Saccharomyces cerevisiae* pretreated with α-phenacyl chloride	167.7	3.5	87.5	1.5% ethanol (v/v)	[30]
Lyophilized cells of *E. coli* BL21/pCgKR 2 and lyophilized GDH powders	1000	140.1	>99	27% glucose	[10]
Whole cells of *E. coli* BL21 coexpressing IolS and GDH	50	3.8	99.5	3.6% glucose	[31]
Whole cells of *E. coli* BL21 coexpressing IolS and GDH	1600	132.1	99.5	20% glucose	[11]
d-LDH from *Staphylococcus epidermidis* and FDH from *Candida boidinii* *	182	38.2	>99.8	2.2% ammonium formate	[15]
Partially purified d-LDH (EC 1.1.1.28) and whole cells of *Candida boidinii* ATCC 32591 containing FDH*	56.7	49.9	ND	0.8% sodium formate	[12]
Whole cells of *E. coli* BL21 coexpressing YiaE and GDH*	100	4.2	98	3.6% glucose	[1]
Whole cells of *E. coli* DF*	71.8	47.9	>99	0.5% sodium formate	This study

*Substrates were OPBA. Substrates of the other processes were OPBE. ND represents no data.

doi:10.1371/journal.pone.0104204.t003

## Conclusions

In summary, whole cells of *E. coli* DF coexpressing d-nLDH^Y52L/F299Y^ from *L. bulgaricus* ATCC 11842 and FDH from *C. boidinii* NCYC 1513 exhibited catalytic capability for (*R*)-HPBA production from OPBA. After optimization of the biotransformation conditions, 73.4 mM OPBA was reduced to 71.8 mM (*R*)-HPBA with a high productivity of 47.9 mM h^−1^ and an excellent ee (>99%). The constructed coupled biocatalysis system developed in this work may be a promising alternative for the production of the key medical intermediate (*R*)-HPBA.

## Supporting Information

Figure S1 **HPLC analysis of the product of the catalytic reaction by using whole cells of** ***E. coli*** **D2 (A) as the biocatalyst and glucose as the substrate for NADH regeneration or whole cells of** ***E. coli*** **DF (B) as the biocatalyst and sodium formate as the substrate for NADH regeneration.**
(TIF)

Figure S2 **HPLC analysis of the product of the catalytic reaction utilizing the whole cell biocatalyst.** (A) HPLC analysis of (*R*)-HPBA and (*S*)-HPBA. (B) Product of the catalytic reaction.
(TIF)
