# Supplementary figures and images for: Efficient Production of (R)-2-Hydroxy-4-Phenylbutyric Acid by Using a Coupled Reconstructed d-Lactate Dehydrogenase and Formate Dehydrogenase System
Source: PLoS One. 2014 Aug 4;9(8):e104204. doi: 10.1371/journal.pone.0104204 (PMC4121320; doi:10.1371/journal.pone.0104204)

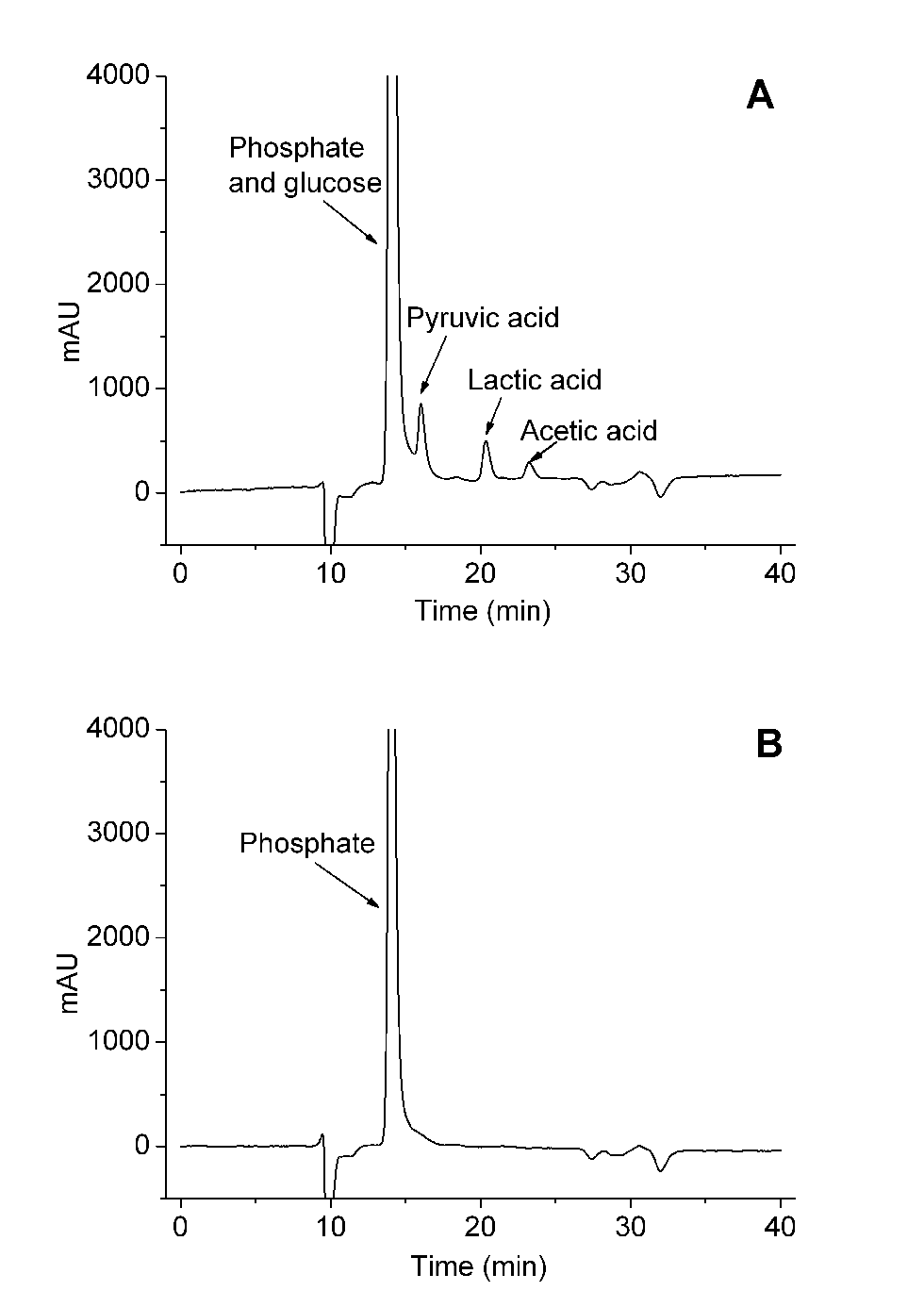

Supplement: Figure S1 — HPLC analysis of the product of the catalytic reaction by using whole cells of E. coli D2 (A) as the biocatalyst and glucose as the substrate for NADH regeneration or whole cells of E. coli DF (B) as the biocatalyst and sodium formate as the substrate for NADH regeneration. (TIF) [file pone.0104204.s001.tif]

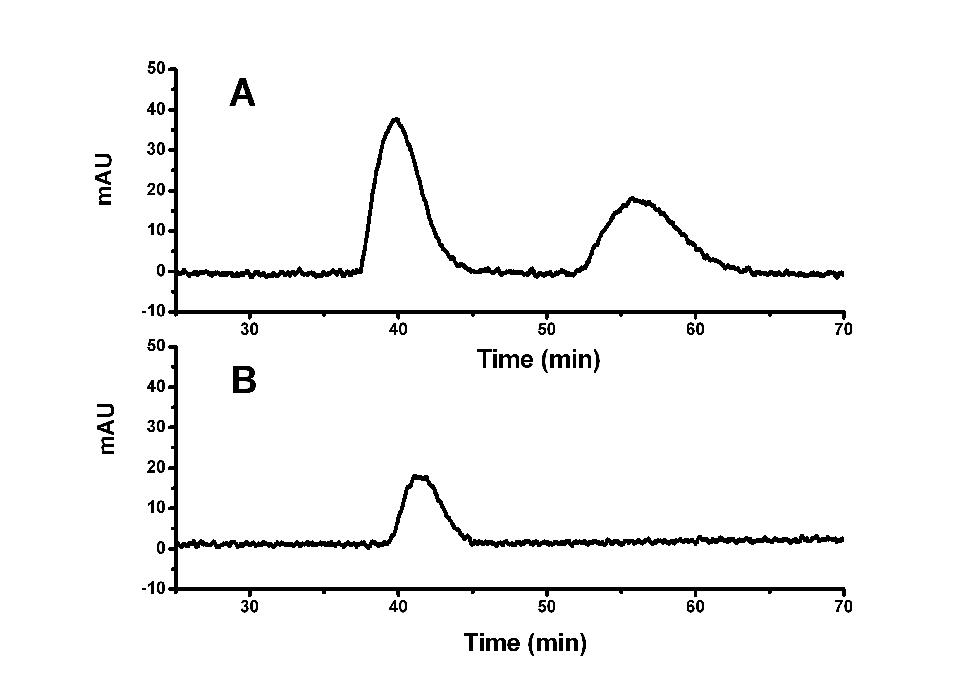

Supplement: Figure S2 — HPLC analysis of the product of the catalytic reaction utilizing the whole cell biocatalyst. (A) HPLC analysis of (R)-HPBA and (S)-HPBA. (B) Product of the catalytic reaction. (TIF) [file pone.0104204.s002.tif]
